# Effects of Fireworks Burning on Air Quality during the Chinese Spring Festival—Evidence from Zhengzhou, China

**DOI:** 10.3390/toxics12010023

**Published:** 2023-12-25

**Authors:** Xinzhan Liu, Ling Yang, Yan Wang, Pengfei Yan, Yimeng Lu

**Affiliations:** 1 College of Geography and Environmental Science, Henan University, Kaifeng 475004, China; lxztt@henu.edu.cn (X.L.); w-yan@henu.edu.cn (Y.W.); 104753220190@henu.edu.cn (P.Y.); 1813040010@vip.henu.edu.cn (Y.L.); 2 Key Laboratory of Geospatial Technology for Middle and Lower Yellow River Regions, Henan University, Ministry of Education, Kaifeng 475004, China

**Keywords:** spring festival, fireworks burning, air quality, pollutants

## Abstract

Fireworks burning significantly degrades air quality over a short duration. The prohibition of fireworks burning (POFB) policy of 2016 and the restricted-hours fireworks burning (RHFB) policy of 2023 in Zhengzhou City provide an ideal opportunity to investigate the effects of such policies and of fireworks burning on air quality during the Spring Festival period. Based on air quality ground-based monitoring data and meteorological data for Zhengzhou City, the article analyzes the impact of the POFB policy and the RHFB policy on air quality. The results show that: (1) The ban on fireworks burning significantly affects Spring Festival air quality, with a decrease of 16.0% in the Air Quality Index (AQI) value in 2016 compared to 2015 and a 74.9% increase in 2023 compared to 2022. (2) From 2016 to 2022, the Spring Festival period witnessed a substantial decrease in average concentration of main pollutants, along with a delayed occurrence of peak concentrations, indicating a noticeable “peak-shaving” effect. However, in 2023, there was an increase in pollutant concentrations, volatility, and a significant surge in hourly concentration. (3) The POFB policy and RHFB policy notably impacted PM_2.5_ and PM_10_, with a decrease of 16.1% and 23.6% in PM_2.5_ and PM_10_ concentrations, respectively, in 2016 compared to 2015, but an increase of 74.5% and 79.2%, respectively, in 2023 compared to 2022. (4) The contribution of fireworks burning to PM_2.5_ concentrations significantly decreased during the fireworks burning period (FBP) in 2016 after the POFB policy and increased significantly in 2023 during FBP after the implementation of the RHFB policy. Unfavorable meteorological conditions will undoubtedly exacerbate air quality pollution caused by fireworks burning.

## 1. Introduction

Fireworks burning is a traditional Spring Festival custom, which has been part of the celebration for more than 1000 years in China. Throughout ancient times, the widespread practice of fireworks burning emerged within Chinese culture. Fireworks are emblematic of peace and prosperity, not only in China but also in other cultures, such as during Diwali in India [1,2,3,4], the Chaharshanbe-Suri Cultural Festival in Iran [5], New Year in the Netherlands [6], and various New Year’s celebrations observed in numerous countries [7,8,9,10,11].

Fireworks are primarily composed of black powder [12], which includes sulfur powder (S), potassium nitrate (KNO_3_), charcoal powder (C), and may also contain potassium chlorate (KClO_3_) and potassium perchlorate (KClO_4_). To achieve vibrant colors, additional components, such as magnesium powder, iron powder, aluminum powder, antimony powder, and inorganic salts, are incorporated into the manufacturing process. Unfortunately, fireworks burning leads to multiple forms of pollution, including noise pollution [13,14,15], light pollution, and significant amounts of litter. Furthermore, fireworks release substantial quantities of particulate pollutants and gas pollutants, such as SO_2_ and NO_x_ (nitrogen oxides), which significantly contribute to the deterioration of air quality. This release affects the health and safety of individuals. Even when weather conditions are favorable for dispersion, the act of fireworks burning can still result in rapid and significant short-term rises in levels of air pollutants [16,17,18,19]. These increases can further exacerbate existing pollution levels if weather conditions are unfavorable, thereby presenting risks to both human health and environmental quality [17,18,19,20,21,22,23,24]. 

The impact of fireworks burning on air quality during the Spring Festival period has garnered significant attention from the academic community. Rindelaub et al. [25] compared PM_10_ samples before and after fireworks burning and found that setting off fireworks and firecrackers caused an increase in airborne particulate matter concentrations and an increased risk of PM_10_ exposure for the population. Moreno et al. [26] collected aerosol samples before and after fireworks burning, and comparative analyses revealed significant increases in concentrations of suspended particulate matter and metals. The study also highlighted significant health risks associated with highly concentrated fireworks burning. Scholars analyzed the effect of fireworks burning on air quality and found that fireworks increased the concentration of aerosol, resulting in the deterioration of air quality with elevated concentrations of main pollutants [1,16,27,28,29,30]. By monitoring the changes in particulate matter in Nanning, Li et al. [31] found that fireworks burning was the main source of particulate matter. Kong et al. [32] also discovered that fireworks burning was the primary source of PM_2.5_, leading to significant increases in heavy metal concentrations and contributing to increased air pollution. Yuan et al. [33] measured the size of aerosols in Chengdu during the Spring Festival period in 2019 and found that fireworks burning leads to enhanced hygroscopicity of aerosols of larger sizes, further exacerbating air pollution in the Sichuan Basin. In their study, Song et al. [34] employed diverse exposure models to compare and analyze the risks associated with PM_2.5_ exposure among residents at various times throughout the year, aiming to assess their health levels. The findings indicated that air quality is notably polluted by fireworks burning. Hickey et al. [35], through a comparison of the metal content in various types of fireworks and their effects on human health, identified a positive correlation between the rise in cardiovascular disease incidence and fireworks emissions. Their research emphasized the imperative of prohibiting fireworks and offered guidance for the development of more environmentally friendly alternatives. In their comparison of air pollution exposure preceding the onset of different types of cardiovascular diseases, Cecconi et al. [36] demonstrated that short-term exposure to air pollutants emitted from a large number of fireworks-related activities significantly increased the probability of myocardial infarction. 

Drawing from an extensive body of research literature, Lin [15] demonstrated the imperative of stringent environmental regulations and holiday restrictions on fireworks burning. Wang et al. [37], through a study on the variations in PM_2.5_ concentration in the air during holiday celebrations, particularly focusing on changes in the concentration of heavy metal substances, confirmed that the intake of heavy metals through respiration can elevate the burden on blood circulation. These substances can then reach various organs through the bloodstream, leading to disorders and dysfunctions in several systems within the human body. Li et al. [38] integrated long-term PM_2.5_ ground monitoring data with volunteers’ blood data to investigate the impact of varying environmental health levels on human blood health. The results confirmed a close correlation between various blood parameters and the level of air pollution. The findings consistently demonstrated a strong association between blood data and air pollution levels. Fan et al. [39] precisely modeled changes in air quality levels in economically developed cities in China, utilizing data from a high-density air observation network of sites to quantify the extent of the impacts. Simultaneously, they proposed the production of more environmentally friendly fireworks in future campaigns to counteract the negative impacts on human health caused by fireworks emissions, leading to significant improvements.

To solve the serious problem of air pollution, government departments have formulated a series of policies, namely no-burn, no-setting off, and no-sale. Scholars conducted a comparative assessment of the air quality before and after the enforcement of the POFB policy and found that the concentrations of particulate showed a decreasing trend after the implementation of the POFB policy, indicating a significant improvement in air quality. Liu et al. conducted a comparative analysis of air quality in Zhengzhou before and after the implementation of the POFB policy spanning from 2014 to 2019. The study revealed a significant “peak-shaving” effect, with findings strongly suggesting that the enforcement of the POFB policy played a crucial role in enhancing air quality in Zhengzhou [40]. Zhao et al. analyzed the impact of fireworks burning on air quality in Linyi City from 2021 to 2022 during the implementation of the POFB policy. Their study focused on the Spring Festival period and revealed a significant improvement in air quality due to the POFB policy. The findings underscored the necessity of implementing the POFB policy during the Spring Festival [41]. Yao et al. analyzed the changes in air quality before and after fireworks burning by measuring the concentrations of gaseous pollutants in Shanghai from 2013 to 2017 and found that PM_2.5_ concentrations showed a decreasing trend after the implementation of the POFB policy [42]. Yang et al. analyzed the changes in PM_2.5_ concentrations before and after the implementation of the POFB policy based on a random forest model and found that restricting fireworks burning had a significant effect on mitigating air pollution and that the air pollution mitigation effect had significant spatial and temporal heterogeneity [43]. Singh et al. found that a government ban on fireworks burning during the COVID-19 period resulted in effective pollution prevention and control during Diwali [44]. Parra et al. modeled changes in PM_2.5_ concentration and observed a decrease, with regards to an earlier fireworks display [14].

The above studies are based on the premise of the POFB policy to explore the overall impact of fireworks burning on the region. However, fewer studies conduct pairwise comparisons before and after the implementation of the RHFB policy. Additionally, existing studies often have a relatively short duration, typically limited to one year. Consequently, long-term comparative serial studies are deficient, and there is no comparative analysis of air quality during the three distinct periods of “no ban—ban—restricted hours” for fireworks burning discharge.

In 2014, Zhengzhou City, the capital of Henan Province, experienced poor air quality, leading to its inclusion in the list of cities with poor air quality made by China’s ecological and environmental monitoring agency [45]. Consequently, the POFB policy was introduced in 2016, with the banning area gradually expanding until 2019, when fireworks were banned citywide in Zhengzhou. However, the municipal administration of Zhengzhou announced its intention to permit limited-duration fireworks burning at the end of December 2022. As a result, in 2023, Zhengzhou witnessed not only extensive fireworks organized on a large scale but also numerous fireworks ignited by individual residents. The implementation of the “no ban—ban—restricted hours” policy on fireworks provides us with a different perspective, making it even more crucial to consistently pay attention to and assess the effectiveness of these prohibition and restriction policies.

By analyzing ground-based air quality monitoring data, we studied the effects of the POFB policy and the RHFB policy on air quality during the fireworks burning period (FBP) and non-fireworks burning period (NFBP) from 2015 to 2023. The results of the study will provide scientific guidance for government.

## 2. Materials and Methods

### 2.1. Study Area

**Figure 1:**
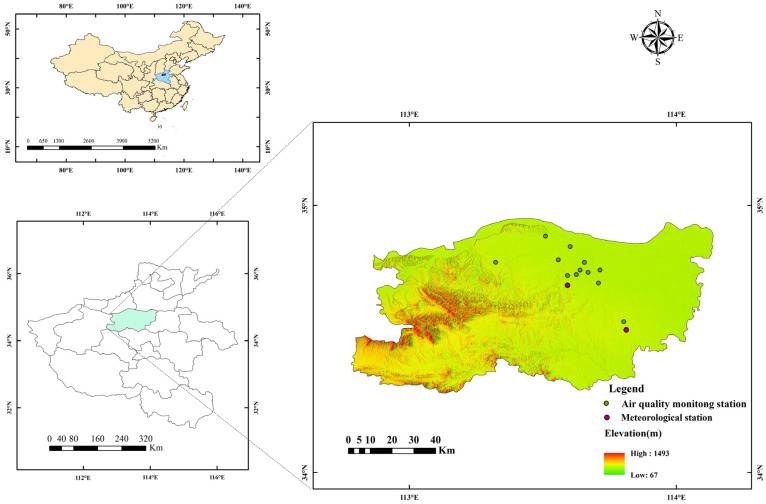
Study area and monitoring stations in Zhengzhou City.

Zhengzhou City (112°42′ E to 114°14′ E, 34°16′ N to 34°58′ N) is located in the central part of Henan province and is the provincial capital. The study area is depicted in Figure 1.

### 2.2. Data Sources

China’s Ministry of Ecology and Environment has implemented a ground-level air quality monitoring system that covers a majority of the country’s cities. The monitoring data are released on its official website. The ground-level air quality monitoring data used in this study are sourced from the China Ecological Environment Monitoring Platform (https://www.air.cnemc.cn:18007, accessed on 3 May 2023). The meteorological monitoring data, which includes information on wind speed and relative humidity, were obtained from the World Weather website “https://www.rp5.ru (accessed on 4 May 2023)”. Figure 1 illustrates the distribution of 12 air quality ground monitoring stations and 2 meteorological stations in Zhengzhou City.

### 2.3. Methods

#### 2.3.1. Data Processing and Analysis

The investigation period spans from the Chinese New Year to six days after the Chinese New Year (from the 1 January to the 7 January in the lunar calendar), comprising a total of 7 days annually from 2015 to 2023.

The following definitions are applied in this study: “daily average” refers to the average of the 24-h concentrations in a day, while “annual Spring Festival average” represents the arithmetical average of the daily concentrations averaged over the entire Chinese Spring Festival period within a calendar year [46]. Following the National Ambient Air Quality Standards, as presented in Table 1, the AQI is divided into six levels, each corresponding to the respective level of air pollution [46,47,48,49].

The pandas package in Python was used for data processing and analysis. The matplotlib package in Python was used for data graph drawing. The air quality monitoring data and meteorological data have passed both the Shapiro–Wilk test and the Levene test. The data meet the necessary criteria for conducting research and analysis.

#### 2.3.2. Correlation Analysis

Pearson’s correlation coefficient is employed to quantify the strength of a linear correlation between two variables. The range of the correlation coefficient is (−1, 1). It is calculated as follows:

(1) $$P_{X,Y}=\frac{cov(X,Y)}{\sigma_{X}\sigma_{Y}}=\frac{n\sum_{i=1}^{n}x_{i}y_{i}-\sum_{i=1}^{n}x_{i}\sum_{i=1}^{n}y_{i}}{\sqrt{n\sum_{i=1}^{n}x_{i}^{2}-(\sum_{i=1}^{n}x_{i}}{)}^{2}\sqrt{n\sum_{i=1}^{n}y_{i}^{2}-(\sum_{i=1}^{n}y_{i}}{)}^{2}}$$

#### 2.3.3. Relative Ratio Method

To exclude the effects caused by meteorological conditions, CO [41,50,51] was selected as a reference indicator to compare and analyze the contribution of fireworks burning to PM_2.5_ before and after the POFB policy and RHFB policy, and calculated as follows:

(2) $$PM_{2.5}(R)=CO\times {\left(\bar{PM_{2.5}}/\bar{CO}\right)}_{P_{nf}}$$

(3) $$PM_{2.5}(F)=PM_{2.5}(M)-PM_{2.5}(R)$$

(4) $$PM_{2.5}(C)=PM_{2.5}(F)/PM_{2.5}\times 100\%$$

The ${\left(\bar{PM_{2.5}}/\bar{CO}\right)}_{P_{nf}}$ represents the ratio of hourly average *PM*_2.5_ to *CO* concentrations during NFBP. The *PM*_2.5_(*R*) represents the effect of other emission sources on PM_2.5_ during FBP. The *PM*_2.5_(*M*) represents the hourly average concentrations of PM_2.5_ during FBP (ug/m^3^). The *PM*_2.5_(*F*) the relationship of fireworks burning to ambient PM_2.5_, with units of μg/m^3^. The *PM*_2.5_(*C*) represents the contributions rate of ambient PM_2.5_, with units of %.

According to the traditional Chinese Spring Festival custom [52,53], the NFBP was defined as 19:00 on Lunar 29 December to 12:00 on NewYear’s Eve, and the FBP was defined as 19:00 on NewYear’s Eve to 12:00 on Lunar 1 January.

## 3. Results

### 3.1. Air Quality Index(AQI) Characteristics

#### 3.1.1. Variation of Annual Spring Festival AQI Average

**Figure 2:**
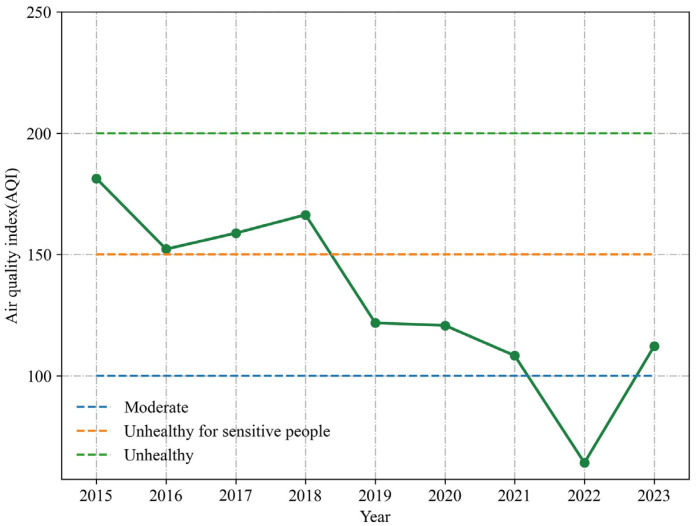
Variation of annual Spring Festival AQI average in Zhengzhou from 2015 to 2023.

As shown in Figure 2, the annual Spring Festival AQI average exhibited a fluctuating pattern, wherein it first decreased sharply and subsequently fluctuated downwards and then saw a sharp rise. The lowest annual Spring Festival AQI average occurred in 2022 (AQI = 64.1), falling under Grade II (Moderate), while the highest was recorded in 2015 (AQI = 181.3), classified as Grade IV (Very unhealthy). This is primarily attributed to Zhengzhou’s initiation of the POFB policy in 2016, resulting in a substantial decrease in fireworks burning. As a result, the annual Spring Festival AQI average in 2016 decreased by 16.0% compared to 2015.

There was a small increase in AQI values in 2017–2018, compared with the same period in 2015. This was largely attributed to the exemption from the RHFB policy for certain areas during the 2017–2018 Spring Festival period. This situation was further exacerbated by a significant number of firework thefts and subsequent illegal discharges [17].

The expansion of the ban on fireworks burning in 2019 led to a significant drop in the AQI average compared to 2018, and the air quality improved overall. From 2019 to 2022, the annual Spring Festival AQI average continued to decrease, due to the POFB policy. The implementation of the RHFB policy in 2023 (AQI = 112.2) resulted in a significant increase in the annual Spring Festival AQI average, rising by 74.9% compared to 2022 (AQI = 64.1), with air quality once again reaching Grade III (Unhealthy for sensitive people) pollution levels.

#### 3.1.2. Variation of Daily AQI Average

**Figure 3:**
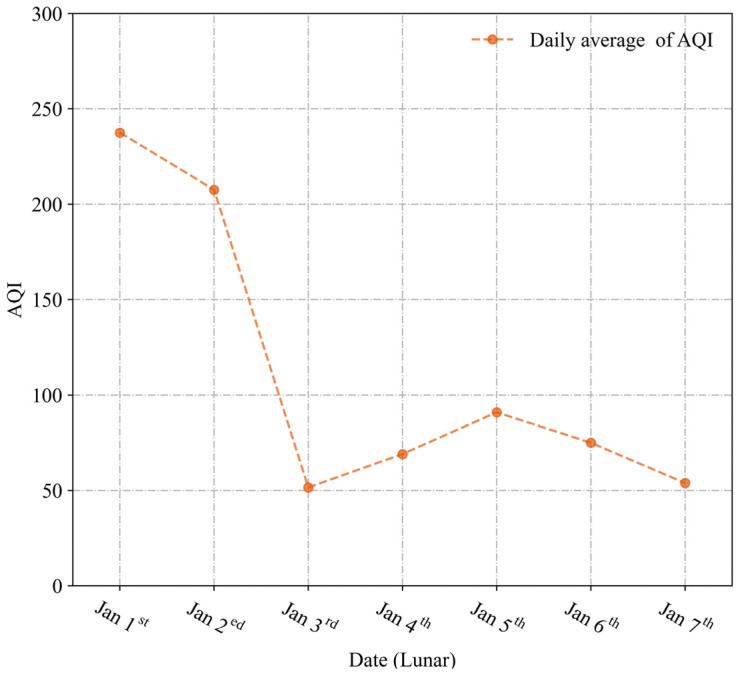
Variation of daily average AQI during the Spring Festival period in 2023.

In 2023, the daily AQI average in Zhengzhou exhibited fluctuations during the Spring Festival period, as revealed in Figure 3. Specifically, the daily average AQI value was 112.2, with the highest values recorded on 1 January and the lowest on 3 January, with values of 237.5 and 51.5, respectively. Notably, the AQI average increased and decreased significantly from 1 January to 3 January, reaching its peak on 1 January.

Such observations can be attributed to the temporary deterioration of air quality resulting from widespread fireworks burning during the Spring Festival period, followed by a continued decrease in existing pollutants leading to an overall improvement in air quality. Essentially, the AQI average displayed noteworthy fluctuations throughout the Chinese Spring Festival period, characterized by single peaks, with the worst air quality recorded on January 1st, followed by a rapid improvement in air quality post-festival.

**Figure 4:**
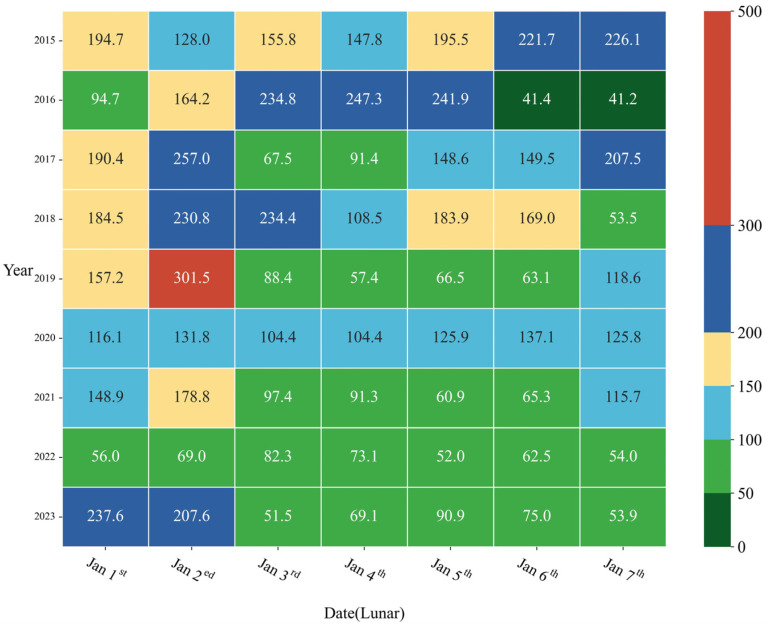
The daily average AQI and corresponding air quality classes for the Chinese Spring Festival period from 2015 to 2023.

Figure 4 presents the daily average AQI and corresponding air quality classes for the Chinese Spring Festival period from 2015 to 2023. From 2017 to 2022, the air quality improved, indicating the effectiveness of the POFB policy in maintaining stable air quality. The peak AQI average on 2 January 2019 was mainly due to elevated humidity and the proximity of Zhengzhou City to the Beijing–Tianjin–Hebei region. The regional transport and diffusion of pollutants from New Year’s Eve fireworks burning also played a contributing role, aligning with the findings of Fu et al. [20].

Conversely, the implementation of the RHFB policy in 2023 resulted in a pronounced degradation of air quality, marked by a notable increase in the AQI average. This can primarily be attributed to residents participating in fireworks activities more frequently due to the policy, resulting in a decline in air quality.

### 3.2. Variation of Concentrations in Main Pollutants

**Figure 5:**
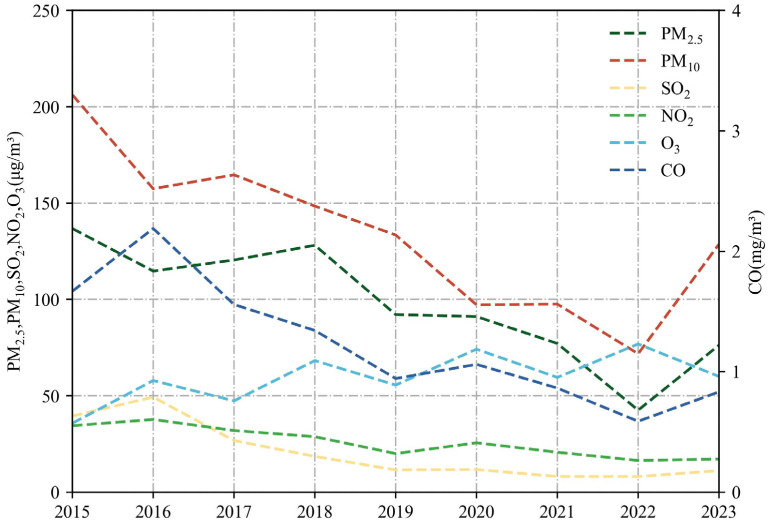
Variation in annual Spring Festival average concentration of main pollutants from 2015 to 2023.

**Figure 6:**
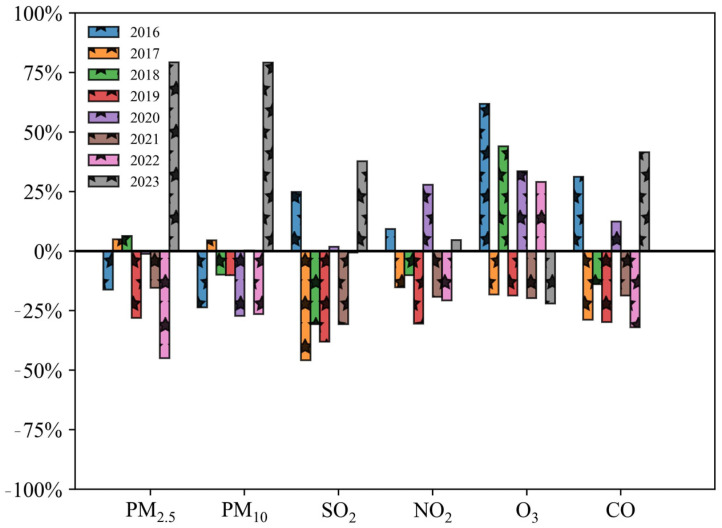
The annual average Spring Festival average of main pollutants showing year−to−year variations from 2016 to 2023.

**Figure 7:**
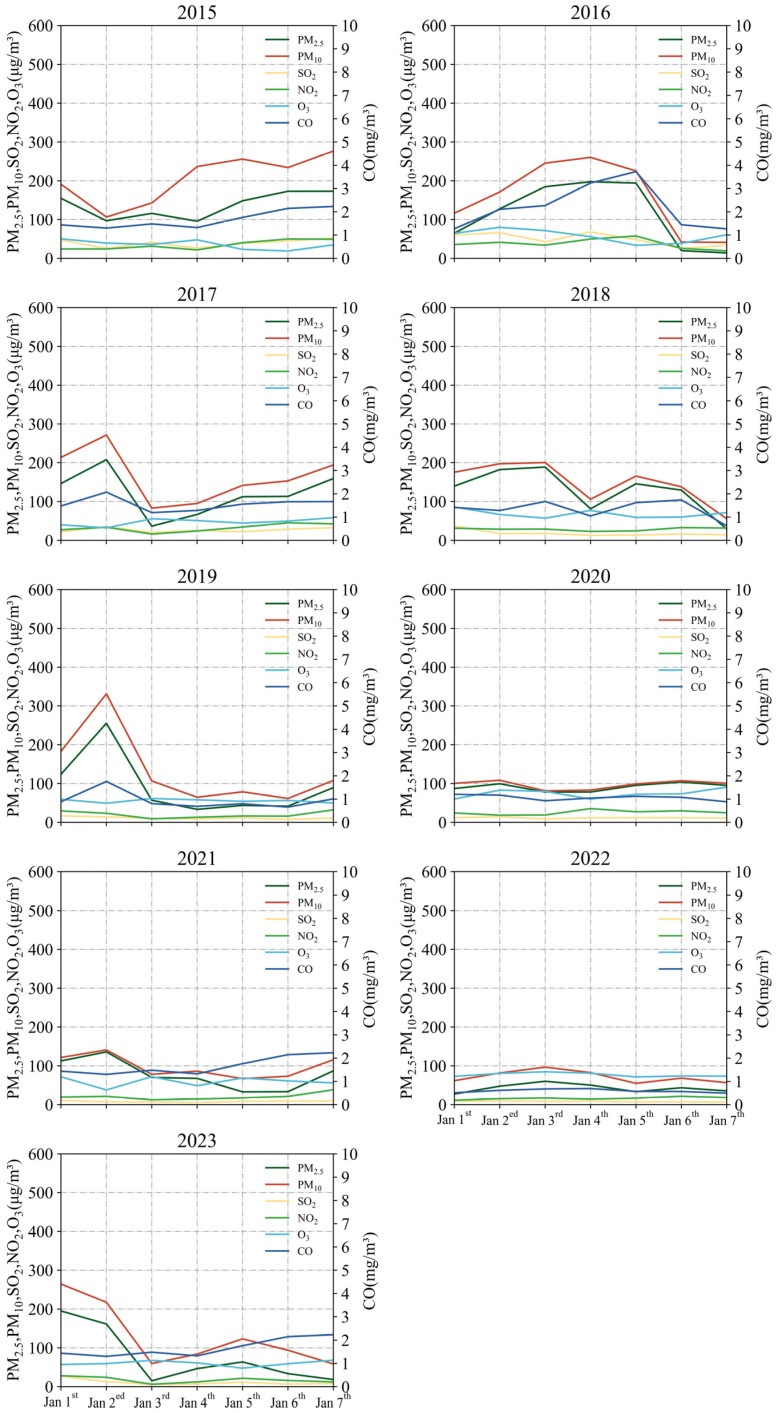
Daily changes in concentrations of main pollutants from 2015 to 2023.

The burning of fireworks has an impact on the levels of different pollutants in the air. Figure 5, Figure 6 and Figure 7 display the changes in average concentrations of the main pollutants from 2015 to 2023, both annually and daily. The concentrations of the main pollutants were discussed in two time periods: first, from 2015 to 2016, which included the period before and after the POFB policy in 2016; second, from 2022 to 2023, which included the period before and after the implementation of the RHFB policy in 2023.

#### 3.2.1. Variation before and after Implementation of POFB Policy (2015–2016)

Based on the graphs in Figure 5, Figure 6 and Figure 7, it is observed that there are changes in the average concentrations of main pollutants. The concentrations of PM_2.5_, PM_10_, SO_2_, NO_2_, O_3_, and CO recorded in 2015 were 136.8, 206.2, 39.5, 34.4, 35.7 μg/m^3^, and 1.6 mg/m^3^, respectively. In 2016, these concentrations were 114.7, 157.4, 49.3, 37.7, 57.9 μg/m^3^, and 2.2 mg/m^3^. Notably, PM_2.5_ and PM_10_ levels significantly declined, while O_3_ concentrations showed a minor increase. Other pollutants remained relatively unchanged during the Spring Festival period from 2015 to 2016.

According to Figure 7, daily average concentrations of PM_2.5_, PM_10_, and O_3_ decreased from 1 January to 2 January 2015. On 2 January, the daily average concentrations of PM_10_, SO_2_, and CO reached their lows at 106.5, 25.5 μg/m^3^, and 1.3 mg/m^3^, respectively. In contrast, PM_2.5_, NO_2_, and O_3_ showed a marked decrease. The daily average concentrations of PM_2.5_ and NO_2_ reached their respective troughs of 95.7 and 21.7 μg/m^3^ on 4 January. Following the implementation of the POFB policy, the daily average concentrations of all pollutants, except O_3_, reached their maximum from 4 January to 5 January 2016. However, there was no notable rise in pollutant concentrations on 1 January 2016.

In conclusion, there was a significantly higher concentration of main pollutants on 1 January before the implementation of the POFB policy. After the introduction of the POFB policy, the daily average concentrations of main pollutants peaked and then rapidly minimized, indicating a significant improvement in air quality due to the POFB policy. The POFB policy has been identified as having the most notable impact on the concentrations of PM_2.5_ and PM_10_, as indicated in previous studies [54,55].

#### 3.2.2. Variation of before and after Implementation of RHFB Policy (2022–2023)

The annual Spring Festival average concentrations of PM_2.5_, PM_10_, SO_2_, NO_2_, O_3_, and CO in 2022 were 42.5, 71.8, 8.0, 16.4, 76.8 μg/m^3^ and 0.5 mg/m^3^, respectively. In 2023, these concentrations were 76.2, 128.6, 11.1, 17.1, and 60.0 μg/m^3^, 0.8 mg/m^3^, respectively.

Compared to 2022, PM_2.5_, PM_10_, SO_2_, and CO exhibited an increase in average concentrations by 79.2%, 79.2%, 37.7%, and 41.5%, respectively. This rise is mainly attributed to the implementation of the RHFB policy, causing an increase in residents’ fireworks activities and subsequently causing a significant decline in air quality.

From 2 January to 4 January 2022, the daily average concentrations of PM_2.5_, PM_10_, SO_2_, and O_3_ and CO peaked at 60.1, 96.6, 9.2, 84.7 μg/m^3^, 0.6 mg/m^3^, respectively. On 1 January, the daily average concentrations of NO_2_, and PM_2.5_ reached a minimum of 11.4, 27.3 μg/m^3^, respectively, while PM_10_ reached a sub-minimum of 61.9 μg/m^3^. This improvement is attributed to the implementation of the POFB policy, contributing to better air quality.

In 2023, the daily average concentrations of PM_2.5_, PM_10_, SO_2_, NO_2_ and CO peaked at 195.0, 264.3, 26.7, 28.1 μg/m^3^, and 1.2 mg/m^3^, respectively, on 1 January. From 1 January to the 3rd, the daily average concentrations of PM_2.5_, PM_10_, SO_2_, NO_2_, CO exhibited a decreasing trend. Furthermore, on 3 January, the daily average concentrations of PM_2.5_, SO_2_, NO_2_, and CO reached their respective trough levels of 15.2, 5.4, 6.1 μg/m^3^, and 0.4 mg/m^3^, with the second trough for PM_10_ recorded at 59.6 μg/m^3^ on the same day in 2023. Following the implementation of the RHFB policy in 2023, the annual Spring Festival average concentrations of main pollutants increased in 2023 compared to 2022.

Additionally, the peak daily average concentration increased, and it occurred significantly earlier, peaking on 1 January. These findings further support the idea that fireworks burning can significantly deteriorate air quality over a brief period.

### 3.3. Variation of Hourly Concentrations of Main Pollutants

**Figure 8:**
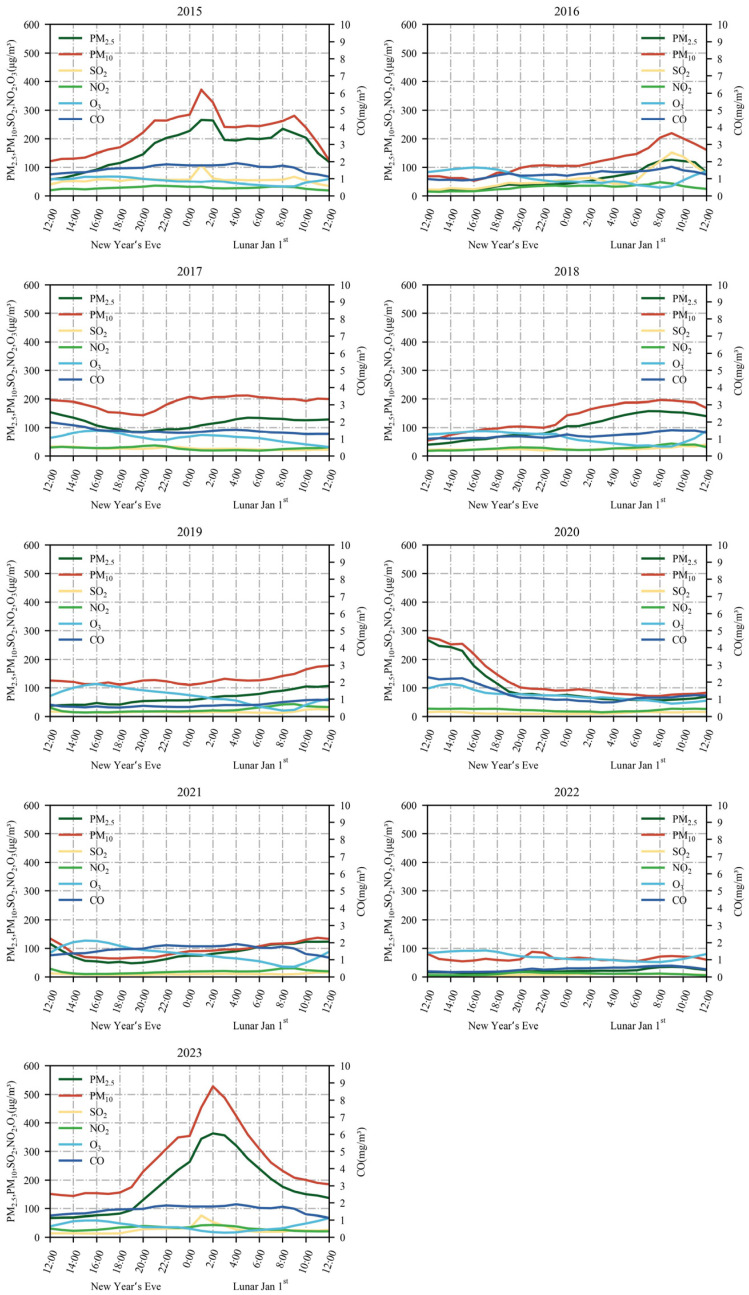
Hourly changes in concentrations of main pollutants from New Year’s Eve to 1 January from 2015 to 2023.

Figure 8 illustrates the variation in hourly concentrations of main pollutants from New Year’s Eve to Lunar 1 January, spanning from 2015 to 2023. As depicted in Figure 8, in 2015, the hourly concentrations of the main pollutant increased from 12:00 on New Year’s Eve, reaching their respective peaks between 0:00–4:00 on 1 January, and then declined afterward. In comparison to 2015, the highest concentration levels of main pollutants were significantly reduced from New Year’s Eve to Lunar 1 January from 2016 to 2022, aligning with the findings of Zhang et al. [56]. These positive outcomes can be attributed to the inhibitory impact of the POFB policy on the elevated pollutant concentration phase.

There was minimal variation in the concentrations of O_3_ from New Year’s Eve to Lunar 1 January from 2015 to 2023. Except for 2017, which displayed a bimodal change, O_3_ concentration increased on the morning of Lunar 1 January in all other years. This phenomenon is closely related to photochemical reactions [57].

Compared to 2022, there has been an increase in the peak hourly concentrations of main pollutants (excluding O_3_) in 2023. This trend can be attributed to the implementation of the RHFB policy, along with a rise in anthropogenic firework activities. Notably, there are similarities between the hourly pollutant concentration trends observed in 2015 and 2023.

### 3.4. Contributions of Fireworks Burning to PM_2.5_ Concentrations during the FBP

**Figure 9:**
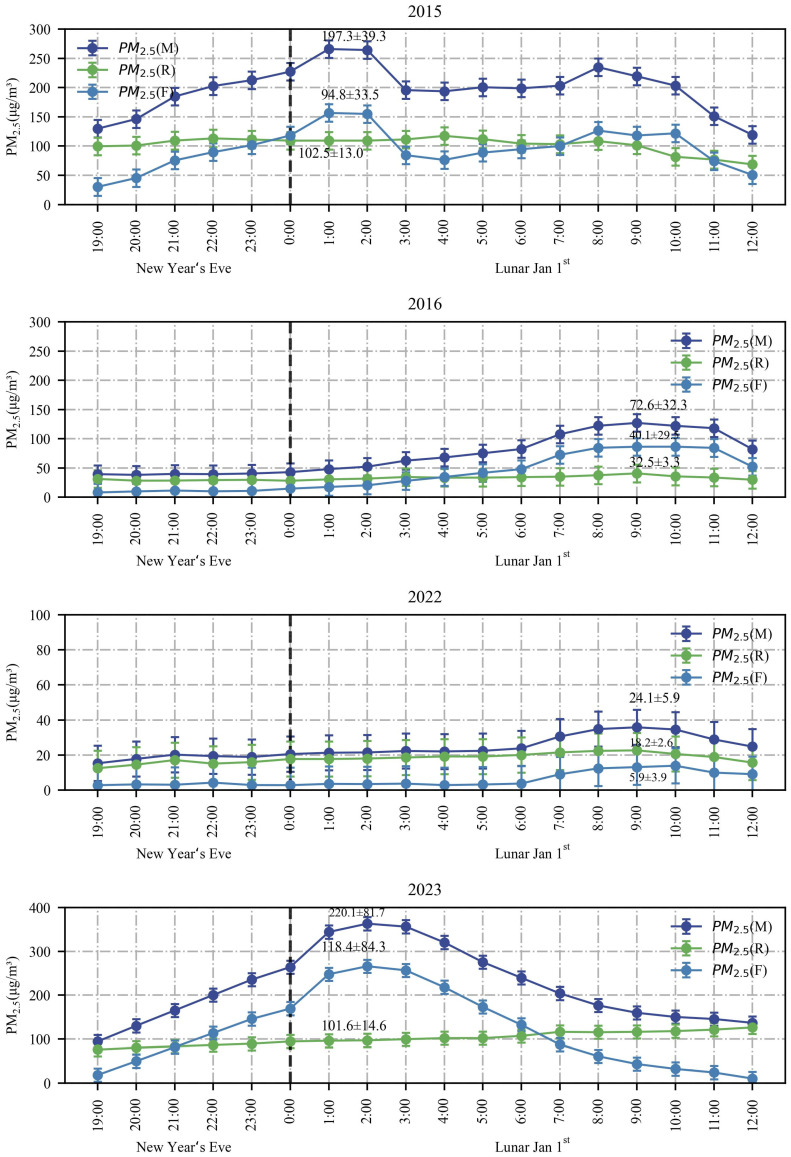
Variation of hourly average contributions of fireworks burning to ambient PM_2.5_ during FBP from 2015 to 2016 and 2022 to 2023.

**Figure 10:**
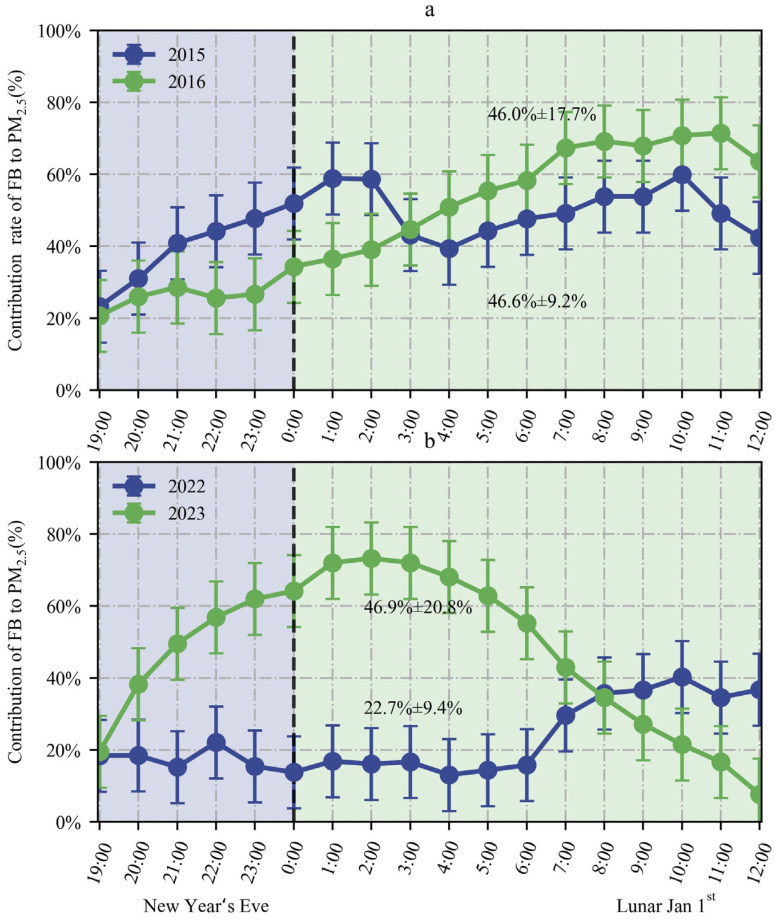
Variation of hourly average contributions rate of fireworks burning to ambient PM_2.5_ in Zhengzhou during FBP from 2015 to 2016 and 2022 to 2023. The average contribution rate of firework activities to PM_2.5_ concentrations during FBP from 2015 to 2016 (**a**); The average contribution rate of firework activities to PM2.5 concentrations during FBP from 2022 to 2023 (**b**).

Figure 9 presents the hourly changes in *PM*_2.5_(*R*), *PM*_2.5_(*M*), and *PM*_2.5_(*F*) concentrations during FBP from 2015 to 2016 and from 2022 to 2023. Figure 10 presents the hourly changes in the contributions of fireworks burning to ambient PM_2.5_ during FBP from 2015 to 2016 and 2022 to 2023.

As illustrated in Figure 9, air quality in Zhengzhou during the Fireworks Burning Period (FBP) in 2016 exhibited improvement compared to 2015, attributed to a reduction in fireworks burning. The average PM_2.5_ concentration during the FBP in 2016 showed a significant decrease, dropping from 97.3 ± 39.3 μg/m^3^ to 72.6 ± 32.3 μg/m^3^, representing a notable decrease of 63.1%. There was a notable decrease in the contribution concentration of fireworks burning to PM_2.5_ during FBP from 2015 to 2016, declining from 94.8 ± 33.5 μg/m^3^ in 2015 to 40.1 ± 29.5 μg/m^3^ in 2016, indicating a decrease of 57.7%.

As illustrated in Figure 10a, compared to 2015, the average contribution rate of firework activities to PM_2.5_ concentrations during FBP in 2016 did not change significantly. However, the contribution rate of firework activities to PM_2.5_ concentration during the period from New Year’s Eve 19:00 to 1 January 3:00 still showed a significant decrease.

As shown in Figure 9, a substantial increase in the concentration of fireworks burning to PM_2.5_ was observed during FBP from 2022 to 2023, rising from 5.9 ± 3.9 μg/m^3^ in 2022 to 118.4 ± 84.3 μg/m^3^ in 2023, indicating an increase of 1884%. Compared to 2022, the average PM_2.5_ concentration during FBP in 2023 significantly increased, from 24.1 ± 5.9 μg/m^3^ to 220.1 ± 81.7 μg/m^3^, reflecting an increase of 810%.

As illustrated in Figure 10b, in comparison to 2022, the average contribution rate of fireworks burning to ambient PM_2.5_ concentrations in 2023 increased significantly, rising from 22.7% ± 9.4% in 2022 to 46.6% ± 20.8% in 2023, indicating an increase of 105%. In 2023, the contribution rate of firework activities to PM_2.5_ exhibited a continuous upward trend (19.4%) from 19:00 on New Year’s Eve, reaching its peak level (73.2%) at 2:00 on Lunar 1 January, remaining at a high level (55.2–73.2%) until 6:00, and then sharply decreasing to 61.2% within 4 h. This observation aligns with the results reported by Kong et al. [32].

Overall, compared to 2015, the contribution of fireworks burning to PM_2.5_ concentrations significantly decreased during FBP in 2016 after the implementation of the POFB policy. Compared to 2022, the contribution of fireworks burning to PM_2.5_ concentrations and the contribution rate of fireworks burning to PM_2.5_ increased significantly during the FBP in 2023 after the implementation of the RHFB policy. This serves as another confirmation of the necessity of administrative prohibitions, which make a crucial contribution to the improvement of air quality.

## 4. Discussion

**Figure 11:**
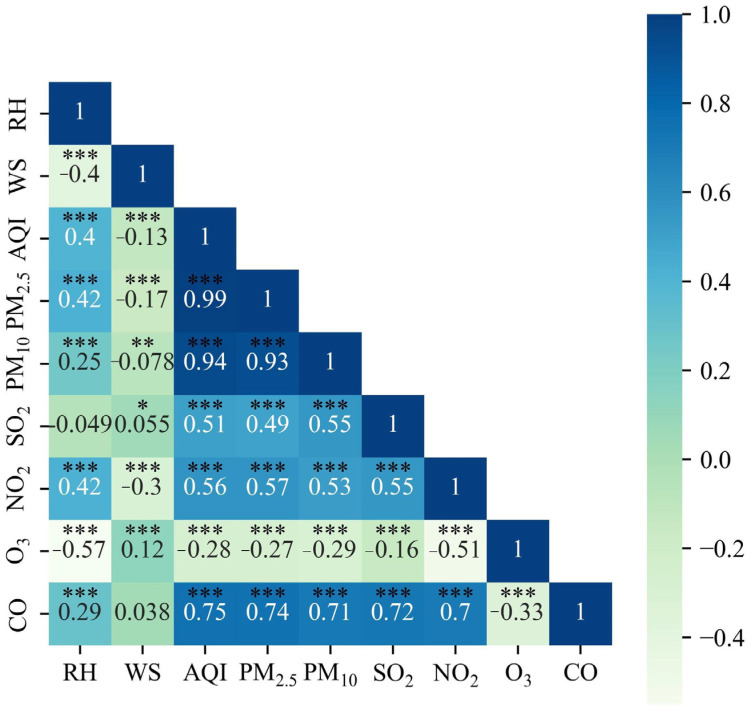
Correlation coefficients between concentrations of main pollutants and meteorological factors. * represent *p* < 0.05, ** represent *p* < 0.01, *** represent *p* < 0.001.

**Figure 12:**
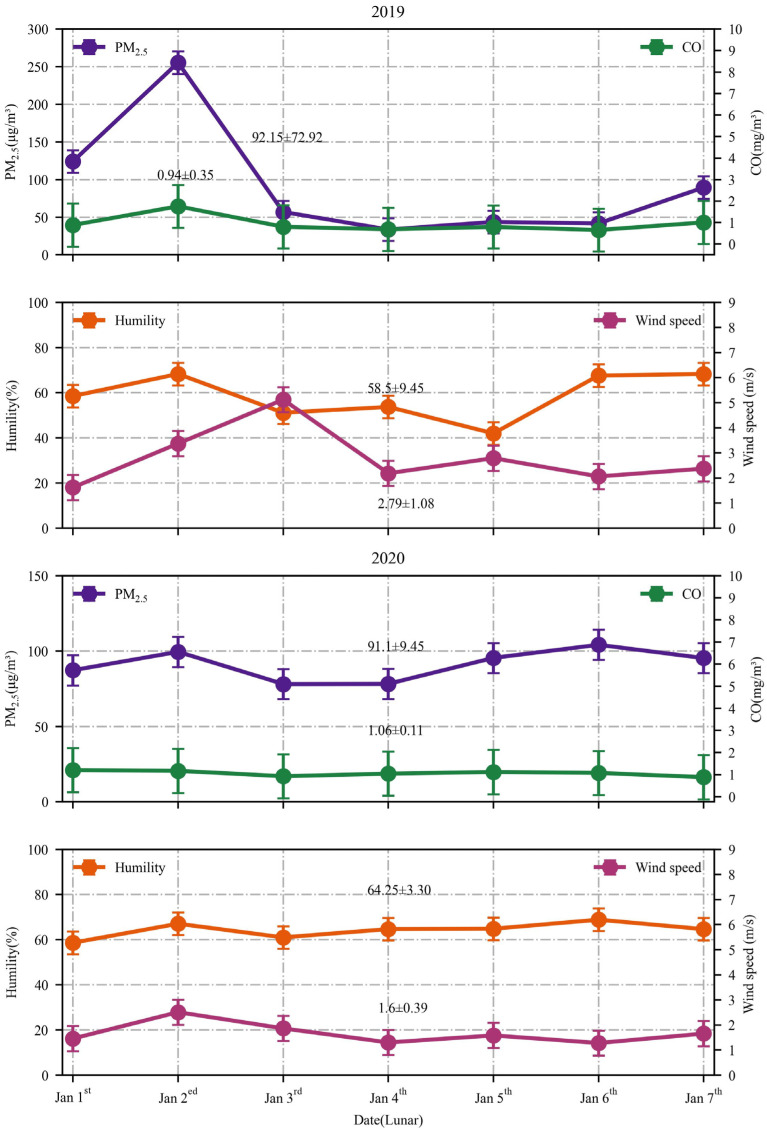
Variation of the daily average of PM_2.5_, CO, relative humidity, and windspeed during the Spring Festival period from 2019 to 2020.

Variations in meteorological parameters can influence air pollution. To further investigate the sudden rise in pollutant levels, the relationship between air pollution and various meteorological factors, including relative humidity (RH) and wind speed (WS), was examined. The meteorological data collected from 2015 to 2023 were analyzed to evaluate the fluctuation pattern, and the association between meteorological factors and the concentrations of main pollutants was assessed. Figure 11 illustrates the correlation coefficients between the hourly average concentrations of main pollutants and meteorological factors. Figure 12 illustrates the variation in the daily average of PM_2.5_, CO, RH, and WS from 2019 to 2020.

As shown in Figure 11, the results depicted reveal a positive correlation between relative humidity and PM_2.5_, PM_10_, NO_2_, CO, and AQI, with correlation coefficients of 0.42, 0.25, 0.42, 0.29, and 0.4, respectively. Increased humidity in winter enhances the moisture absorption capacity of particulate matter, and higher air humidity facilitates the conversion of gaseous pollutants into particulate matter, while also increasing pollutant concentrations [45,51,58]. The wind speed was inversely correlated with AQI, PM_2.5_, PM_10_, and NO_2_.

Fireworks burning activities in 2020 were significantly reduced due to the COVID-19 pandemic. However, the change in average AQI values in 2020 is minimal compared to 2019. Several studies have been conducted to demonstrate that variations in meteorological parameters can influence air pollution [59,60,61].

As shown in Figure 12, compared to 2019, the average PM_2.5_ concentration and the average CO concentration in 2020 did not change significantly. However, compared to 2019, the annual Spring Festival average wind speed decreased significantly by 42.65% and the annual Spring Festival average humidity increased significantly by 9.8% in 2020. The change in wind speed and relative humidity caused a smaller change in the annual Spring Festival AQI average in 2020 compared to 2019. The local air quality is influenced by the transmission and diffusion of air pollutants from neighboring areas, an aspect that will be further explored in future studies.

## 5. Conclusions

This study comprehensively analyzed the effects of fireworks burning on the concentrations of main pollutants. The primary objective was to assess the impact of both the POFB and RHFB policies on air quality. The key conclusions drawn from the study are as follows.

Firstly, the POFB policy had a significant positive impact on air quality in Zhengzhou.

In 2016, the first year of the ban, AQI values notably improved (AQI = 152.2) compared to 2015 (AQI = 181.3), reflecting a 16.0% decrease. Continued implementation of the POFB policy led to remarkable air quality improvements in 2022, with an AQI average of 64.1. However, air quality deteriorated significantly after the implementation of the RHFB policy. In 2023, the first year of the RHFB policy, the situation worsened considerably (AQI = 112.2) compared to 2022 (AQI = 64.1), with a 74.9% increase in AQI values.

Secondly, the ban effectively mitigated the abrupt increase in pollutant concentration and delayed the occurrence of peak values, demonstrating a noticeable “peak shaving” effect. Throughout the introduction of the POFB policy, there was a consistent decrease in the annual Spring Festival average concentration of pollutants, a decline in the peak value of daily average concentration, and a reduction in the rate of change. Conversely, after the introduction of the RHFB policy, the annual Spring Festival average concentration and daily average concentration of pollutants increased significantly, with a more evident increase in amplitude and enhanced fluctuation.

Thirdly, the implementation of the POFB policy had the most noticeable impact on the concentration of PM_2.5_ and PM_10_. In 2016, when burning was prohibited, the concentrations of PM_2.5_ and PM_10_ decreased by 16.14% and 23.64% respectively, compared to 2015. In contrast, in 2023, when burning was not forbidden, the was an increase of 79.7% and 79.2%, respectively, compared to 2022. Compared to 2015, the contribution of fireworks burning to PM_2.5_ concentrations significantly decreased during FBP in 2016 after the POFB policy. Compared to 2022, the contribution of fireworks burning to PM_2.5_ concentrations and the average contribution rate of fireworks burning to PM_2.5_ increased significantly during the FBP in 2023 after the implementation of the RHFB policy.

Lastly, unfavorable meteorological conditions will undoubtedly exacerbate air quality pollution caused by fireworks burning, impeding the dilution and diffusion of pollutants, and contributing to significantly worse air quality.

## Figures and Tables

**Table 1:** AQI values, Grade, and Air Quality.

Air Quality	AQI Value	Grade
Good	0–50	I
Moderate	51–100	II
Unhealthy for sensitive people	101–150	III
Unhealthy	151–200	IV
Very healthy	201–300	V
Hazardous	301–500	VI

## Data Availability

Data are available upon request to the corresponding author.
